# Parameter Optimization and DEM Simulation of Bionic Sweep with Lower Abrasive Wear Characteristics

**DOI:** 10.3390/biomimetics8020201

**Published:** 2023-05-13

**Authors:** Shuo Wang, Xuanting Liu, Tianjian Tong, Zihe Xu, Yunhai Ma

**Affiliations:** 1 The College of Biological and Agricultural Engineering, Jilin University, 5988 Renmin Street, Changchun 130025, China; 2 The Key Laboratory of Bionic Engineering, Ministry of Education, Jilin University, 5988 Renmin Street, Changchun 130025, China; 3 Department of Agriculture and Biosystems, Iowa State University, Ames, IA 50011, USA

**Keywords:** biomimetic design, DEM, abrasive wear, Archard wear, sweep

## Abstract

High wear rates during the tillage process often result in significant financial losses and wasted farming seasons. In this paper, a bionic design was used to reduce tillage wear. Inspired by wear-resistant animals with ribbed structures, the bionic ribbed sweep (BRS) was designed by combining a ribbed unit with a conventional sweep (CS). BRSs with different parameters (width φ, height h, angle θ, and interval λ) were simulated and optimized using the DEM and RSM methods at a working depth of 60 mm to evaluate the magnitude and trends of three responses: tillage resistance (TR), number of contacts between the sweep and soil particles (CNSP), and Archard wear value (AW). The results showed that a protective layer could be created on the surface of the sweep with a ribbed structure to reduce abrasive wear. Analysis of variance proved that factors φ, θ, and λ had significant effects on AW, CNSP, and TR, while factor h was insignificant. An optimal solution was obtained using the desirability method, including 8.88 mm φ, 1.05 mm h, 3.01 mm λ, and 34.46° θ. Wear tests and simulations showed that wear loss could be effectively reduced at different speeds by the optimized BRS. It was found to be feasible to create a protective layer to reduce partial wear by optimizing the parameters of the ribbed unit.

## 1. Introduction

As agricultural technology has progressed, the wear failure of tillage has worsened [1]. Nearly 60% of financial losses have been attributed to missed production time due to part replacement and maintenance [2]. Of these wear failures, abrasive wear accounts for up to 50% [3]. Low-stress abrasion occurs when soil-engaging components come into direct contact with soil particles during the tillage process. This can erode the edges and surfaces of parts, consuming more energy and reducing product quality [4]. The problems caused by wear are extremely serious; therefore, scholars have used various methods to increase the wear resistance of tillage components.

Previous studies have focused on decreasing abrasive wear by increasing the surface hardness of tillage parts. Approaches to improving wear resistance include the use of high-strength materials [5], heat treatment [6,7], and the application of hardfacing materials to the base metal [8,9].

All of these techniques can significantly extend the lifespan of tools. One of the most effective methods of creating new materials is the bionic approach. Natural organisms have developed remarkable abilities to adapt to their environments over hundreds of millions of years of evolution. Some plants and animals have surfaces that are consistently structured and shaped reasonably [10,11]. The tamarisk and scorpion that survive in sandy environments, the sea conch that lives in a highly abrasive mud environment, and the mole shell that lives in soil have all served as sources of inspiration. Tian, et al. [12] investigated the microstructures of shell surfaces to understand their anti-wear mechanisms. Su, et al. [13] found that mole shells were covered with ribs capable of resisting variable natural frictional erosion. Similarly, Huang, et al. [14] and Yin, et al. [15] discovered that the grooved structures on tamarisk surfaces could provide erosion resistance. Some researchers have created samples with ribbed or grooved surfaces with varying widths, spacings, and angles to study anti-wear mechanisms [16,17,18].

The discrete element method (DEM), developed by Cundall and Strack [19], is commonly used to simulate dynamic processes. Scholars have utilized this technique in modeling various agricultural engineering processes. These processes include optimizing soil–tool interactions [20,21], estimating tillage disturbance caused by tools [22,23], and simulating grain mixture screening and cleaning processes [24,25]. This technique has proven to be effective in achieving these objectives. DEM can also be used to simulate abrasive wear during tillage. Kalacska, et al. [26] confirmed the wear mechanism by simulating soil particle movement in contact with a tine using DEM. Zhang, et al. [27] simulated the wear behavior of several bionic surfaces using the EDEM Archard wear model and found that the results matched experimental outcomes. Awuah, et al. [28] assessed the geometric and vibrational properties of various tines using DEM, demonstrating its ability to accurately predict component wear and tillage resistance. Tong, Mohammad, Zhang, Ma, Rong, Chen, and Menon [1] investigated how changes in Farrer scallop morphology affect wear patterns. Previous studies did not analyze the combined effect of the bionic unit size system on the wear resistance of tillage components.

The purpose of this study was to use bionics methods to reduce the wear of tillage components caused by soil particles and to focus on the influence of specific parameters on wear resistance. In this investigation, ribbed units were integrated with a conventional sweep (CS) to produce a biomimetic ribbed sweep (BRS), which was designed to replicate the wear-resistant characteristics of shell-like organisms. The DEM was employed to simulate the tillage process. The response surface methodology was utilized to optimize the width, height, intervals, and angles of the ribbed unit in order to create a BRS with optimal wear resistance.

## 2. Materials and Methods

### 2.1. Design of BRS

**Figure 1:**
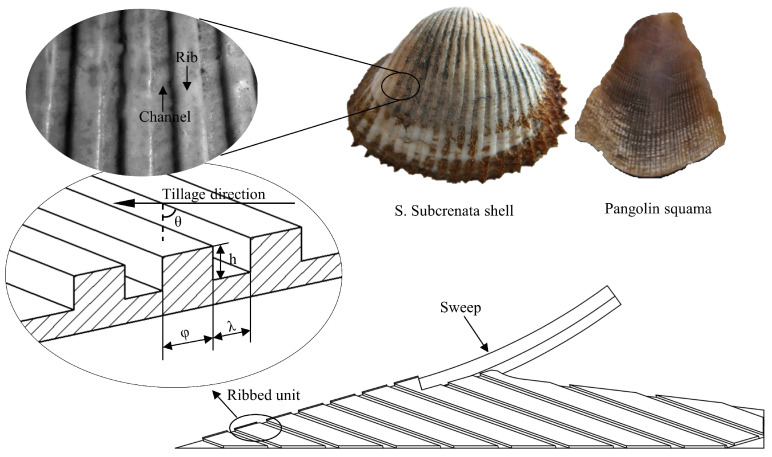
Schematic of biomimetic samples covered with various ribbed units extracted from *S. Subcrenata* [12] and *Pangolin squama* [16].

**Figure 2:**
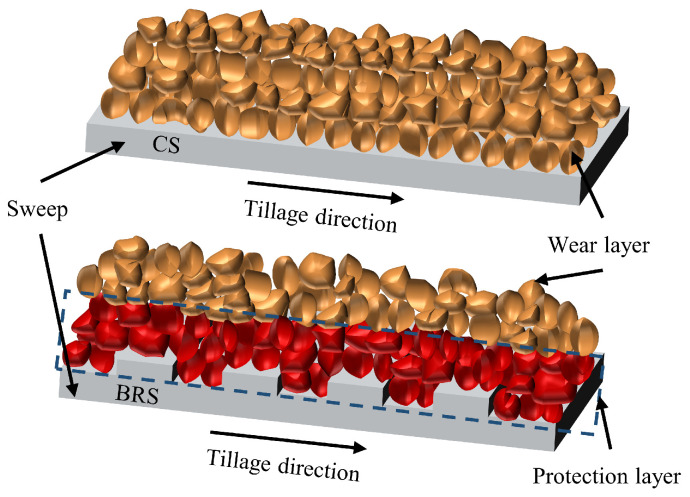
A schematic diagram of the wear reduction mechanism by the ribbed unit.

A conventional sweep (CS), which is commonly used, was modified to incorporate a bionic ribbed unit into the design of a biomimetic ribbed sweep (BRS), as depicted in Figure 1. The design was inspired by the ribbed surface features of shells and pangolins. The primary characteristics of the bionic ribbed unit included the width φ, height h, angle θ, and intervals λ. The presence of ribbed units altered the interaction between soil particles and the sweep surface, resulting in the separation of soil into two layers. As shown in Figure 2, these layers consisted of a protection layer with a small speed difference between soil particles and the sweep, and a wear layer with a large speed difference. This alteration shifted the wear process from being between the soil and sweep to being between the sweep and the protection layer, as well as between the protective and wear layers [15,29].

### 2.2. DEM Simulation

Soil samples were taken from the topsoil layer (0–10 cm) [30] in the experimental field of the Jilin Agricultural University, Changchun, Jilin Province. The soil density was measured to be 1.27 g/cm^3^ and the soil moisture content was determined to be 22.33%.

In this study, the tillage procedure was simulated using EDEM software. The main parameters employed in EDEM included material characteristics, such as density, Poisson’s ratio, and shear modulus, as well as contact property parameters. The Hertz–Mindlin (no slip) contact model was utilized in this investigation and is characterized by three contact coefficients: coefficients of restitution, static friction, and rolling friction. The range of soil parameters was determined based on previous studies. Material and interaction parameters were then adjusted using static angle tests to obtain the appropriate parameters [31,32].

#### 2.2.1. Static Angle Tests and Simulation

**Figure 3:**
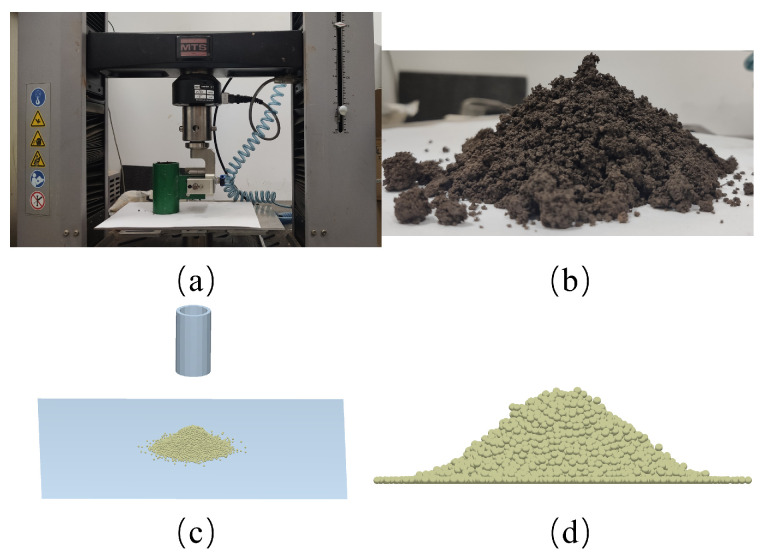
Experiment and simulation of static angle, (**a**) MTS test device, (**b**) test soil pile, (**c**) simulation test device, and (**d**) simulation soil pile.

The static angle test was conducted using a pipe with an inner diameter of 46 mm and a height of 105 mm. The pipe was filled with soil and suspended on the electronic universal testing apparatus (C43.104, MTS Systems Co., Ltd., Canton, OH, USA). Soil particles were released from the pipe to create a stable stack as it was dragged upward at a speed of 10 mm/s. Three replicate tests were carried out and six sets of photos were obtained at 30° increments. The same settings were used to construct the EDEM simulation shown in Figure 3. By collecting screenshots at the same 30-degree intervals as the experiment, six sets of soil stacking photos were obtained in the EDEM. The static angle on both sides of the pile shown in Table 1 was extracted from the collected pictures using MATLAB (MathWorks, Nadik, MA, USA). The results revealed that the experimental mean static angle was 35.69°, with a standard deviation (Std. Dev) of 1.16 and a coefficient of variation (C.V) of 3.25%. The simulation’s average static angle was 35.55°, with a Std. Dev of 1.09 and a C.V of 3.06%. Both the test and simulation C.V values were less than 15%, indicating that the results were properly collected and reliable [33]. The adopted parameters presented in Table 2 were appropriate, as evidenced by the relative error of 0.39% between the test and simulation.

#### 2.2.2. DEM Soil Bin

**Figure 4:**
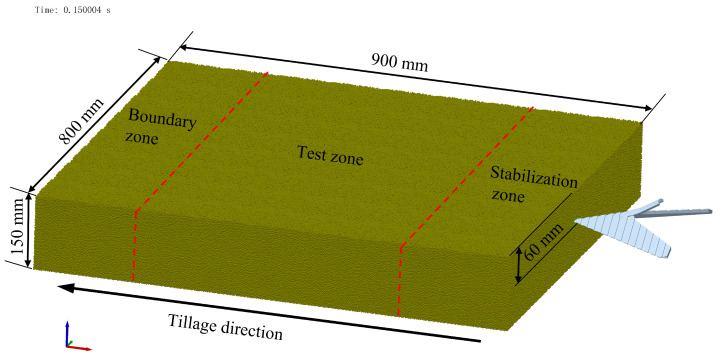
Soil bin and the sweep position in the DEM simulation model.

Figure 4 depicts a virtual soil bin with dimensions of 900 × 800 × 150 mm (long × width × depth). To create the soil bin, spherical particles with a radius of 1.5 mm were used and their sizes were randomized between 0.95 and 1.05 times the original size. The bin contained a total of 3,940,848 particles. In the simulation, the fixed time step was set to be 15% of the Rayleigh time step (2.4426 × 10^−5^ s). A 65 Mn steel material was assigned to the sweep model when it was imported into EDEM, as indicated in Table 2. The tillage speed was set at 1.5 m/s and the depth was set at 60 mm [26,34].

In order to replicate the model’s wear, the Archard wear model was incorporated into the contact setup. A wear factor of 0.8 × 10^−12^ m^2^N^−1^ for NM360 wear-resistant steel was utilized, as this study mainly focused on the impact of the rib unit on wear [28,35].

The soil bin was divided into three zones: the stable zone, the test zone, and the border zone. The simulation results included mean values for tillage resistance (TR), contact number between sweep and soil particles (CNSP), and Archard wear value (AW) in the test zone.

### 2.3. RSM Experimental Design and Optimization

The single-factor pretest yielded approximations for the ranges of four factors: φ value of 5–15 mm, h value of 0.5–2 mm, λ value of 1–10 mm, and θ value of 0–90°. A Box–Behnken experiment with four factors at three levels was conducted using Design-Expert 13.0 software to examine the interactions between the four factors’ impacts on CNSP, AW, and TR. A total of 29 trials were conducted in a random sequence. The three responses were evaluated using the average of the DEM results and analyzed using analysis of variance (ANOVA) to determine the main effects and interacting variables for each response.

To represent the desired ranges for each response, a multiple-response approach was described using the desirability method [36,37]. The approach used the desirability function, as shown in Equation (1). The response value was ranked from least to most favorable based on *D*, which ranged from 0 to 1. In most cases, a range of 0.8 to 1 in factor D was considered acceptable and excellent. When this value was less than 0.63, the parameter quality was regarded as poor [38].

(1) $$D={\left(d_{1}\cdot d_{2}\cdot \ldots \cdot d_{n}\right)}^{\frac{1}{n}}={\left(\prod_{i=1}^{n}d_{i}\right)}^{\frac{1}{n}}$$

where *n* is the number of the response and di represents each response.

The importance of the response (*r_i_*) was determined by modifying the desirability function *D*(x) throughout the optimization process using Design-Expert. The importance (*r_i_*) ranged from 1 for the least important to 5 for the most significant. The objective function was Equation (2) when various importance levels were applied to various responses. The main goal of this study was lower CNSP and to decrease abrasive wear on sweeps. Although adding ribbed units would increase TR, the optimization outcome should not provide an excessively high value. Thus, TR’s importance was set to level 1, while CNSP’s and AW’s importance was set to level 5. Every response goal used the minimal option. This setting ensured that the optimized process could decrease CNSP and AW while decreasing the rise in TR.

(2) $$D={\left(d_{1}^{r_{1}}\cdot d_{1}^{r_{2}}\cdot \ldots \cdot d_{1}^{r_{n}}\right)}^{\frac{1}{\sum r_{i}}}={\left(\prod_{i=1}^{n}d_{i}^{r_{1}}\right)}^{\frac{1}{\sum r_{i}}}$$

### 2.4. Abrasion Wear Test

**Figure 5:**
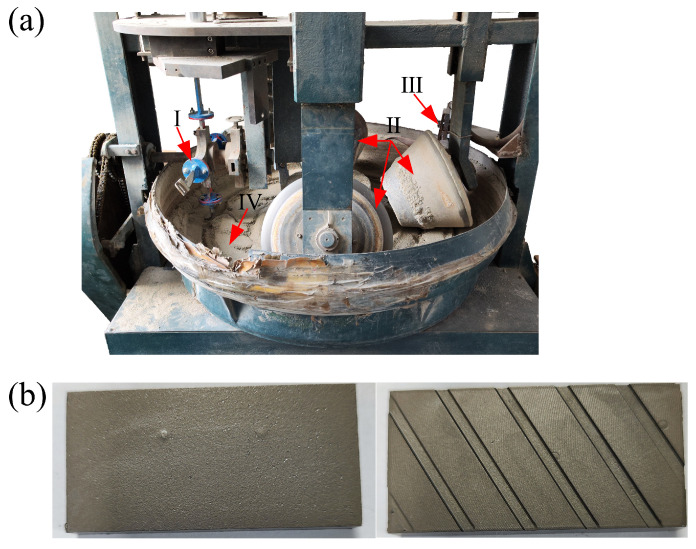
(**a**) JMM abrasive wear tester: (I) Sample holder, (II) Suppression roller, (III) Soil scraper, (IV) Rotary abrasive bin; (**b**) Samples of CS and BRS.

The abrasion wear test was conducted using a JMM abrasive wear tester (designed by Jilin University and the Chinese Academy of Agricultural Mechanization Sciences) to verify the simulation results. As shown in Figure 5, the samples were 60 mm long, 20 mm wide, and 10 mm thick. The distance between the rotary bin’s axis of rotation and the sample’s installation position was 400 mm. The sample’s burial depth was 80 mm and the abrasive’s impact angle on the sample’s surface was 35°. The abrasive used in this study comprised a mixture of 96.5% quartz sand (with a particle size ranging from 0.214–0.420 mm) and 3.5% bentonite (less than 76 μm). Each test set consisted of four samples that were shifted by the rotating system while sliding, resulting in a total distance of 803.4 m per set. As a result, the total wear distance for each set was 25708.8 m. Each sample was tested three times at three different wear speeds: 1.01 m/s, 2.02 m/s, and 3.02 m/s.

## 3. Results

### 3.1. Model Fitting and Checking

Table A1 presents the impact of various BRS parameters on the three responses. Table A2 provides an overview of the evaluation of the sufficiency and applicability of the prediction model for the three responses. The quadratic model was found to be the best prediction model for all responses. The differences between the predicted R^2^ and the adjusted R^2^ were less than 0.2, indicating that the model fit the data well and could be used for interpolation. The model was deemed appropriate, as the lack of fit value for all three responses was not significant.

**Figure 6:**
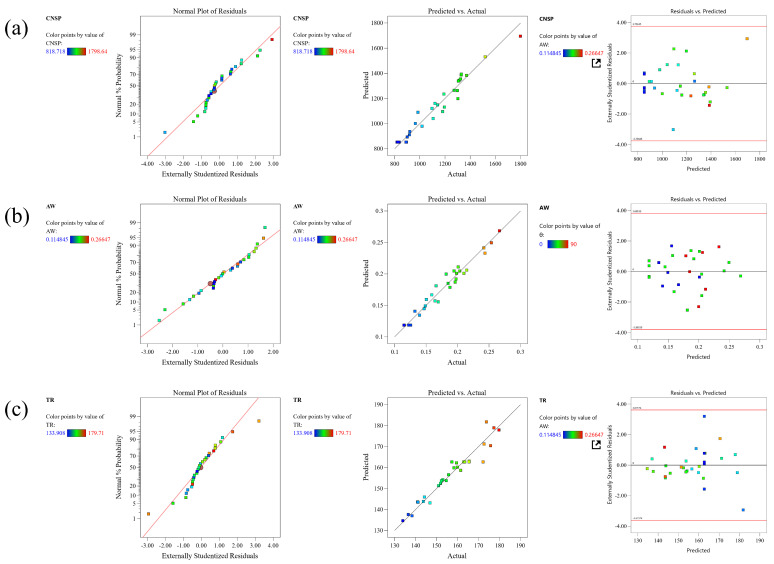
Normal plots of residuals, predicted versus actual plots, and residual vs. predicted plots of three responses: (**a**) CNSP; (**b**) AW; (**c**) TR.

The residual analysis test results of each response-fitting model are shown in Figure 6. The data points in Figure 6 display a normal distribution trend and are dispersed in a straight line. There were fewer unpredictable values in the prediction model due to the smaller discrepancy between the EDEM-simulated data points and the prediction model. As a result, the model employed in this study was deemed reliable. To analyze all responses, the ANOVA method was utilized, and the outcomes are presented in Table A3.

### 3.2. CNSP Prediction

**Figure 7:**
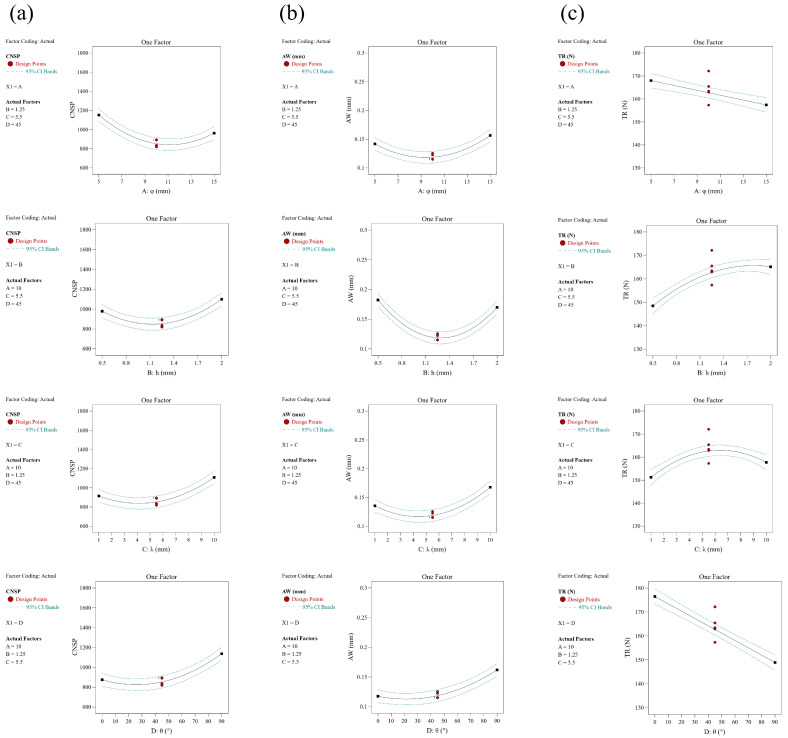
Effect of the single factors on three responses: (**a**) CNSP; (**b**) AW; (**c**) CNSP.

**Figure 8:**
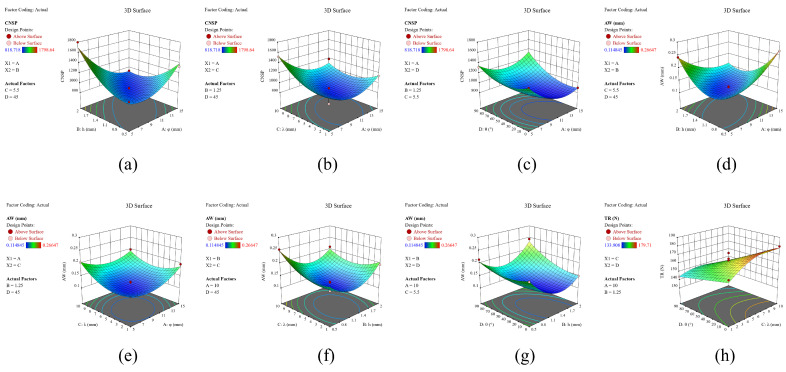
Response surface and contour graphs of different interaction terms: (**a**) Φ and h on CNSP; (**b**) λ and Φ on CNSP; (**c**) θ and Φ on CNSP; (**d**) Φ and h on AW; (**e**) λ and Φ on AW; (**f**) λ and h on AW; (**g**) θ and h on AW; (**h**) λ and θ on TR.

The number of soil particles that came into contact with the sweep during the DEM simulation was defined as CNSP. Its value was related to the magnitude of wear [16]. The DEM simulation results generally increased from 818.72 to 1798.64, with a mean value of 1143.66 in Table A1. The coding factor regression equation is shown in Equation (3). For ease of study, we used A to represent factor φ, B to represent factor h, C to represent factor λ, and D to represent factor θ. Table A3 shows that the main influence factors were *A*, *B*, *C*, *D*, *AB*, *AC, AD, A*^2^, *B*^2^, *C*^2^, and *D*^2^. The CNSP was significantly impacted by the individual factors φ, h, λ, and θ, all of which tended to initially decrease and then increase when each factor increased, as shown in Figure 7a. The interaction terms *AB*, *AC*, and *AD* shown in Figure 8a–c were significant and the contour plots of the response surfaces for *AB* and *AD* were elliptical. This indicates that there was a significant interaction between the component elements in these phrases. When h was at the middle level for factor *AB*, CNSP initially decreased and then increased as φ increased; as h increased, the proportion of CNSP’s decline phase also increased. The interaction effect for factor *AC* was not significant, with an increase in factors φ or λ; CNSP initially decreased and then increased. For factor *AD*, CNSP first decreased and then increased with an increase in φ, and significantly increased with an increase in θ.

(3) $$\begin{array}{l} CNSP=853.18-94.28A+60.20B+97.17C+131.01D-297.74AB\\ -123.51AC+91.28AD+204.37A^{2}+185.82B^{2}+158.82C^{2}+152.99D^{2} \end{array}$$

### 3.3. AW Prediction

AW represented the mean wear value of the sweep model in the EDEM simulation. The simulation results showed a minimum value of 0.114848 mm and a maximum value of 0.114848 mm; the maximum value was 0.26647 mm and the mean value of 0.1774 mm (Table A1). The coding factor regression equation is presented in Equation (4). Table A3 indicates that *A*, *C*, *D, AB*, *AC, BC*, *BD, AD*, *A*^2^, *B*^2^, *C*^2^, and *D*^2^ were significant factors for AW. Although factor B was not significant (*p* = 0.063), it was retained due to its significant interaction term. As shown in Figure 7b, the AW of all single factors initially decreased and subsequently increased with each factor; this trend was similar to that of CNSP. The response surface diagrams for the interaction terms are shown in Figure 8d–g. The contour plots for factors *AB, BC*, and *BD* were distinctly elliptical, indicating significant interactions between them. In factor *AB*, when h was at an intermediate value, AW first decreased then increased as φ increased; the diminishing section of AW slowly grew as h increased from its lowest to highest level. In factor *BC*, AW first decreased then increased as h increased; for factor λ, the response had a similar tendency to h, but with a higher increasing proportion. In terms of *BD*, AW first decreased then increased as h increased, and it considerably increased with θ.


(4) $$\begin{array}{l} AW=0.1186017+0.0074A-0.0062B+0.016C+0.022D-0.048AB\\ -0.014AC-0.018BC+0.030A^{2}+0.058B^{2}+0.033C^{2}+0.021D^{2} \end{array}$$

### 3.4. TR Prediction

TR is one of the major parameters of tillage components. A higher TR requires larger tractors and increases fuel consumption [39,40]. The simulation results ranged from 133.91 to 179.71, with an average value of 156.87 (Table A1). The coding factor regression equation is shown in Equation (5). It can be seen in Table A3 that *A, B, C, D, CD, B*^2^, and *C*^2^ were significant variables. Figure 7c shows the impact of all single factors on TR: it decreased as φ and θ increased, while it first increased and then decreased as h and λ increased. Figure 8h shows that the contour of *CD* was irregularly circular and highly interactive. As λ increased gradually, the value of TR also increased, but started to decrease more dramatically as θ increased.


(5) $$TR=162.70-5.30A+8.35B+3.25C-13.79D-6.36CD-5.90B^{2}-8.20C^{2}$$

### 3.5. Optimization Results

By using the methods described in Section 2.3, the three response prediction models were optimized and the results, are displayed in Table 3. The optimal approach had a desirability rating of 0.925, which was deemed acceptable and excellent. As shown in Table 3, the best result was obtained with the following parameters: 8.88 mm φ, 1.05 mm h, 3.01 mm λ, and 34.46° θ. These parameters produced the best solutions of 0.115 mm AW, 825.88 CNSP, and 159.27 N TR. This produced optimal solutions of 0.115 mm AW, 825.88 CNSP, and 159.27 N TR. The results of the response optimization were suitable and reliable, as they were within the 95% prediction interval, as shown in Table A4. These results could be used to predict the tillage process.

The optimized BRS was re-simulated in EDEM using the same parameters. The results were 0.124 mm AW, 866.64 CNSP, and 146.72 N TR. The relative errors between the simulated value and the predicted value for each case were 7.83%, 4.93%, and 7.88%, respectively. These findings indicate that the results obtained from the regression equation were both accurate and efficient [41]. The simulation results for CS were 0.168 mm AW, 1298.23 CNSP, and 129.66 N TR. Compared with CS, the optimized BRS showed a decrease of 26.19% in AW and a decrease of 33.24% in CNSP, while showing an acceptable increase of 13.16% in TR due to the resistance at a depth of 60 mm being between 129.66 to 179.17 N, which would not have a significant effect on the tillage process. To verify the model’s effectiveness at different tillage speeds, the optimized BRS and CS were simulated in the same soil bin at speeds of 1 m/s, 2 m/s, and 3 m/s. At a speed of 1 m/s, the simulation results for CS were 0.141 mm AW, 1381.87 CNSP, and 116.08 N TR, while the results for the optimized BRS were 0.105 mm AW, 1050.67 CNSP, and 134.52 N TR. At a speed of 2 m/s, the results for CS were 0.196 mm AW, 1218.08 CNSP, and 144.52 N TR, while those for BRS were 0.152 mm AW, 631.27 CNSP, and 164.1 N TR. At a speed of 3 m/s, the results for CS were 0.323 mm AW, 1087.49 CNSP, and 183.99 N TR, while those for BRS were 0.251 mm AW, 514 CNSP, and 210 N TR.

### 3.6. Results of Abrasive Wear Test

**Figure 9:**
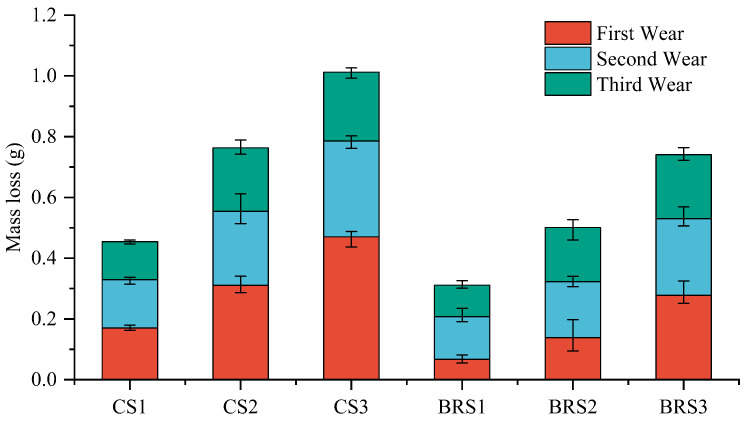
The results of different abrasive speeds, where CS1, CS2, and CS3 represent the wear of CS at 1 m/s, 2 m/s, and 3 m/s, and the same applied to BRS.

The results of the wear test are shown in Figure 9, where CS1, CS2, and CS3 represent the wear of CS at 1 m/s, 2 m/s, and 3 m/s, respectively. The same applied to BRS. As the wear speed increased, the weight loss also increased. The wear loss of the BRS at three speeds decreased by 31.497%, 34.355%, and 26.859%, compared with the CS, respectively. This observation is similar to the findings of Goeke et al. [42], where the loss experienced during the first wear process was greater than that observed in the subsequent wear processes. The reason for this difference was attributed to the sample being in its run-in phase during the initial wear process. During this phase, the surface of the sample was worn down, resulting in an increase in its surface area. At the same time, the wear loss was reduced due to strain hardening, which resulted in the formation of an oxide film on the sample’s surface. The second and third wear processes were in a stable wear phase. Overall, the optimized sweep showed a significant reduction in wear compared with CS at different tillage speeds, which is consistent with the simulation results.

## 4. Discussion

According to this study, the wear characteristics of the sweep were impacted by the ribbed unit. For the CNSP and AW responses, the order of importance for factors was λ, θ, Φ, and h. Parameter h has the least effect, which agreed with the results of Han, et al. [43]. It is feasible to reduce tillage wear by selecting appropriate optimization parameters. In this study, the lowest value of each factor’s range was considered the low level, the highest value was considered the high level, and the midpoint between the low and high levels was considered the medium level. The individual analysis of each factor is described below.

**Figure 10:**
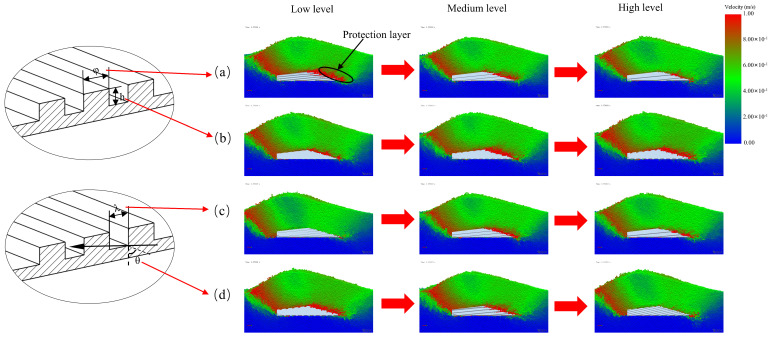
Results of the effect of different factors on particle speed, (**a**) φ, (**b**) h, (**c**) λ, (**d**) θ.

Φ had a significant effect on the CNSP, AW, and TR. Figure 10a shows that the thickness of the soil protection layer gradually decreased as the level increased from low to medium. However, all levels were able to completely enclose the sweep’s surface. At a low level, an increase in the number of ribbed units on the BRS led to an increase in both CNSP and AW due to the increase in the contact area between the BRS and soil. As a result, CNSP, AW, and TR all declined throughout the rise from the low to medium level. The increase in φ from medium to high resulted in an increase in the number of groove units. The soil particle velocity on the surface of the sweep gradually increased. When it reached a certain limit, the protective capacity would be smaller than the increase in wear caused by the increase in area, leading to increases in CNSP and AW. This is consistent with the findings of Zhang, et al. [29], while the values of CNSP and AW steadily increased, and the value of TR decreased throughout this period.

Factor h had a significant effect on CNSP and TR. Although its effect on AW was not significant, it was still investigated for the reasons outlined in Section 3.3. The effect of h on the three responses was primarily due to its impact on the ribbed unit’s ability to hold soil particles. As shown in Figure 10b, at the low level, the surface lacked the ability to hold particles. The soil particle velocity on the surface of the sweep was high, and the protective layer generated was almost imperceptible. This resulted in large CNSP and AW values and poor TR. When h reached a medium level, the velocity on the surface of the sweep decreased and the thickness of the protective layer increased, which was sufficient to protect the sweep. It would lead to a significant decrease in CNSP and AW and an increase in TR. As h increased from the medium to high level, the soil particle velocity on the surface of the sweep decreased and became similar to the tillage speed. Additionally, the protective layer remained adequate to completely cover the sweep’s surface at the intermediate level, which did not affect the CNSP and AW values. However, an increase in the contact area brought about by h led to an increase in both CNSP and AW. In terms of TR, there was no difference between the medium and high levels due to the similar thicknesses of the protective layer.

The influence of λ was found to be significant for all three responses. Its value directly influenced the capacity to form the protective layer. Figure 10c shows that, at low levels of λ, due to the high soil particle velocity on the surface of the blade, there was essentially no protective layer present. As λ increased to a medium level, a consistent protective layer that completely enclosed the sweep formed. This level had the highest TR and the lowest CNSP and AW. However, if λ was increased to a high level, soil particles would erode the inner surface due to the excessive length [17]. Figure 10c shows that the protective layer’s thickness was insufficient to cover the surface of the BRS. Therefore, CNSP and AW were higher at this level than those at the medium level, while TR was lower.

The simulation results for factor θ are presented in Figure 10d. It can be observed that the thickness of the protective layer was sufficient to completely enclose the sweep at low and medium levels. Due to the increased number of ribbed units, both CNSP and AW were higher at a low level than at a medium level. At a high level, however, the protective layer was virtually non-existent. In line with the findings of Tong, et al. [44], CNSP and AW were significantly higher at this level compared with those at the previous two levels. As θ increased, a notable dispersing effect (preventing soil particle accumulation) was observed, while the hindering effect (formation of a protective layer) became negligible. This resulted in a lower TR [18]. These results are consistent with those reported by Zhao, et al. [11], indicating that superior anti-wear performance could be achieved at this angle.

## 5. Conclusions

In this study, a biomimetic method was proposed to reduce a sweep’s wear during tillage by generating a protective layer. The impact of the ribbed unit parameters (φ, h, λ, and θ) on the indicators of CNSP, AW, and TR was investigated using DEM and RSM techniques. BBK tests were performed to obtain the regression equation for each response. The simulation results for the various ribbed parameter scales were analyzed. It was shown that the method for minimizing wear by creating a shield of soil particles was feasible. The regression equations were optimized using the desirability approach, and the final parameters set (8.88 mm φ, 1.05 mm h, 3.01 mm λ, and 34.46° θ) were shown to be the most effective. The relative errors between the predicted and the simulated values were 7.83%, 4.93%, and 7.88%, respectively. The optimized sweep was verified by simulation and testing with three tillage speed. The results show that, compared with the CS, the optimized BRS had a greater reduction in both AW and CNSP. Compared with the CS, the optimized BRS showed a decrease of 26.19% in AW and a decrease of 33.24% in CNSP. It was proven that reducing wear was feasible by optimizing the ribbed unit parameters to create an optimal protective layer. According to the abrasive test, the wear loss of the BRS at three speeds decreased by 31.497%, 34.355%, and 26.859% compared with the CS, respectively. Further research should consider the potential effect of tillage conditions more carefully, for example, the tillage speed, depth, and the diameter of soil particles.

## Figures and Tables

**Table 1:** Simulation and experimental results of static angle test.

Shoot Orientation (deg)	Static Angle of Experiment (deg)	Static Angle of Simulation (deg)
0	36.9833	35.3445
30	34.5819	35.5740
60	34.3060	35.7740
90	34.8940	34.5803
120	37.2428	35.5314
150	36.1415	36.5112
**Parameter**	**Experiment Value**	**Simulation Value**
Static angle (deg)	35.6916	35.5526
Std. Dev (deg)	1.1594	1.0894
C.V (%)	3.2483	3.0643

**Table 2:** Soil and sweep parameters used in the DEM simulation.

Parameters	Soil	Sweep (Steel)
Particle radius (mm)	1.5	
Density of soil particles (kg/m^−3^)	2550	7850
Poisson’s ratio of soil	0.3	0.3
Shear modulus of soil (MPa)	1 × 10^8^	7.9 × 10^10^
Coefficient of restitution (with soil)	0.6	0.5
Coefficient of static friction (with soil)	0.54	0.64
Coefficient of rolling friction (with soil)	0.3	0.2

**Table 3:** Summary of the optimization results.

Φ (mm)	h (mm)	λ (mm)	θ (°)	AW (mm)	CNSP	TR (N)	Desirability (%)
8.88	1.05	3.01	34.46	0.115	825.88	159.27	0.926

## Data Availability

The data presented in this study are available on request from the corresponding author.

## Appendix A

**Table A1:** Response summary of the EDEM simulation result data.

Response	Name	Units	Observations	Minimum	Maximum	Mean	Std. Dev	Ratio
R1	AW	mm	29	0.114845	0.26647	0.1774	0.0429	2.32
R2	CNSP		29	818.718	1798.64	1143.66	232.59	2.20
R3	TR	N	29	133.908	179.71	156.87	12.90	1.34

**Table A2:** Summary of regression model fits for all response variables.

Source	Sequential *p*-Value	Lack of Fit *p*-Value	Adjusted R^2^	Predicted R^2^	Remark
AW model fit summary statistics					
Linear	0.2435	0.0003	0.0625	−0.0927	
2FI	0.2582	0.0003	0.1530	−0.0506	
Quadratic	<0.0001	0.0587	0.9444	0.8479	Suggested
Cubic	0.1217	0.0994	0.9718	0.4004	
CNSP model fit summary statistics					
Linear	0.0550	0.0014	0.1949	0.0580	
2FI	0.0667	0.0023	0.4077	0.3697	
Quadratic	<0.0001	0.1135	0.9298	0.8125	Suggested
Cubic	0.1133	0.2221	0.9654	0.4298	
TR model fit summary statistics					
Linear	<0.0001	0.3292	0.7305	0.6791	
2FI	0.5965	0.2916	0.7148	0.5783	
Quadratic	0.0003	0.9423	0.9136	0.8542	Suggested
Cubic	0.9737	0.5388	0.8440	−0.3190	

**Table A3:** Summary of ANOVA parameters in all responses.

	AW (Quadratic)			CNSP (Quadratic)			TR (Quadratic)	
Source	F-Value	*p*-Value	Source	F-Value	*p*-Value	Source	F-Value	*p*-Value
Model	35.89	<0.0001 ^1^	Model	30.62	<0.0001 ^1^	Model	45.21	<0.0001 ^1^
A	5.67	0.0300 ^1^	A	24.91	0.0001 ^1^	A	24.43	<0.0001 ^1^
B	4.00	0.0629 ^2^	B	10.16	0.0054 ^1^	B	60.56	<0.0001 ^1^
C	27.07	<0.0001 ^1^	C	26.47	<0.0001 ^1^	C	9.16	0.0064 ^1^
D	50.98	<0.0001 ^1^	D	48.11	<0.0001 ^1^	D	165.36	<0.0001 ^1^
AB	80.96	<0.0001 ^1^	AB	82.82	<0.0001 ^1^	CD	11.71	0.0026 ^1^
AC	6.58	0.0208 ^1^	AC	14.25	0.0015 ^1^	B^2^	17.40	0.0004 ^2^
AD	4.19	0.0585 ^2^	AD	7.78	0.0126 ^1^	C^2^	33.64	<0.0001 ^1^
BC	11.24	0.0040 ^1^	A^2^	63.28	<0.0001 ^1^			
BD	13.37	0.0021 ^1^	B^2^	52.32	<0.0001 ^1^			
A^2^	52.02	<0.0001 ^1^	C^2^	38.22	<0.0001 ^1^			
B^2^	185.72	<0.0001 ^1^	D^2^	35.46	<0.0001 ^1^			
C^2^	61.04	<0.0001 ^1^						
D^2^	25.15	0.0001 ^1^						
Lack of fit	4.97	0.0675 ^2^	Lack of fit	3.92	0.0986^2^	Lack of fit	0.3613	0.9387^2^

A: φ, B: h, C: λ, D: θ; ^1^: Statistically significant (*p* < 0.05); ^2^: Statistically not significant (*p* > 0.05).

**Table A4:** Confirmation data for predictive models.

Solution 1	Predicted Mean	Predicted Median	Std Dev	SE Mean	95% CI Low for Mean	95% CI High for Mean	95% TI Low for 99% Pop	95% TI High for 99% Pop
AW	0.114916	0.114916	0.009817	0.004112	0.10615	0.12368	0.07136	0.15847
CNSP	825.877	825.877	65.4314	26.8849	769.155	882.599	543.128	1108.63
TR	159.271	159.271	3.71592	1.16935	156.839	161.703	144.376	174.166
